# Follicular Lymphoma Presenting as Intussusception in an Adult

**DOI:** 10.7759/cureus.16345

**Published:** 2021-07-12

**Authors:** Muhammad S Haq, Daoyi Yang, Yiting Li, Shazia M Shah

**Affiliations:** 1 Internal Medicine, Jersey Shore University (Saint Francis Medical Center), Trenton, USA; 2 Internal Medicine, Saint Francis Medical Center, Trenton, USA

**Keywords:** intussusception, follicular lymphoma, chemotherapy, adults, immunotherapy

## Abstract

A 61-year-old male with no past medical history presented with intense abdominal pain for three days, associated with hematochezia, nausea, and non-bloody vomiting. CT scan of the abdomen showed distended small bowel, diffuse lymphadenopathy, and intussusception of the distal ileum into the cecum with obstruction. Ileocolic resection and histopathological staining confirmed the diagnosis of follicular lymphoma and appropriate treatment was initiated. Intussusception is a condition that involves the invagination of the proximal segment of a bowel tract into its contiguous distal segment as a result of enthusiastic or impaired peristalsis. Only 5% of the total intussusception cases are found in adults. Most cases in adults are caused by pathological lead points which can be benign or malignant. Lymphomas rarely present with intussusception and follicular lymphomas are even less common. To the best of our knowledge, there have only been a few such cases of follicular lymphomas with the initial presentation of intussusception. In this article, we present a rare case of follicular lymphoma presenting as intussusception. Considering lymphomas as a cause of intussusception in adults can decrease diagnostic delays and guide treatment.

## Introduction

Intussusception is a condition that involves the invagination of the proximal segment of a bowel tract into its contiguous distal segment as a result of enthusiastic or impaired peristalsis [1-2]. It is a rare entity in adults and represents only 5% of the total number of cases of intussusception. Unlike in children, intussusception in adults is mostly the result of pathological conditions that function as well-defined lead points. These lead points can be benign, such as polyps, diverticula, benign neoplasms, or malignant neoplasms, which include carcinomas and gastrointestinal (GI) lymphomas, to name a few [1]. Lymphomas can sometimes be present in the GI tract either as an extranodal manifestation or as a primary GI tumor. These lymphomas are extremely aggressive and can sometimes even lead to intussusception [2]. In this article, we aim to highlight an exceedingly rare case of follicular lymphoma presenting as intussusception. Considering lymphomas as a cause of intussusception in adults can decrease diagnostic delays and guide treatment.

## Case presentation

**Figure 1:**
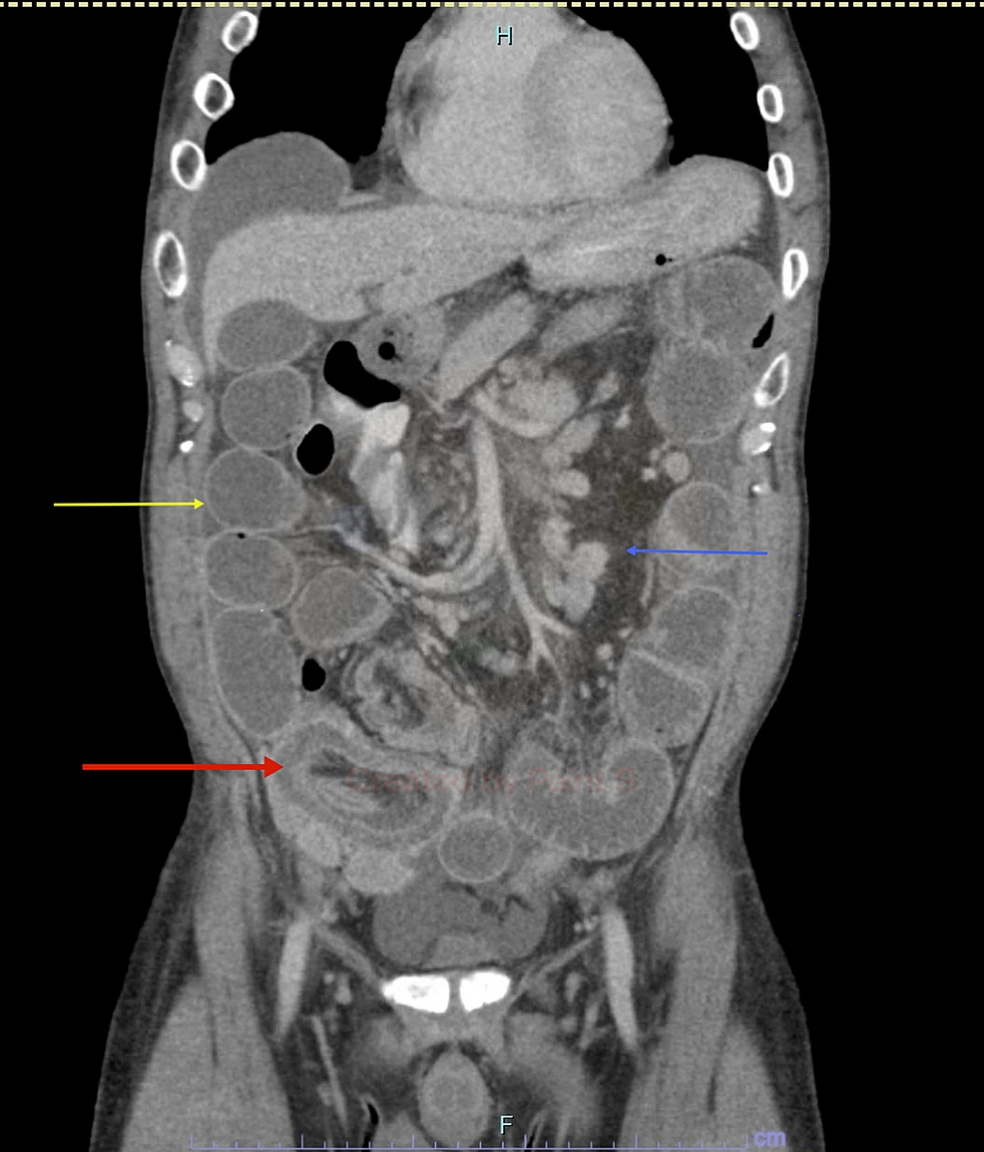
Abdominal CT scan with contrast (coronal view) revealing a distended small bowel (yellow arrow), mesenteric lymphadenopathy (blue arrows), and intussusception of distal ileum into cecum (red arrow).

**Figure 2:**
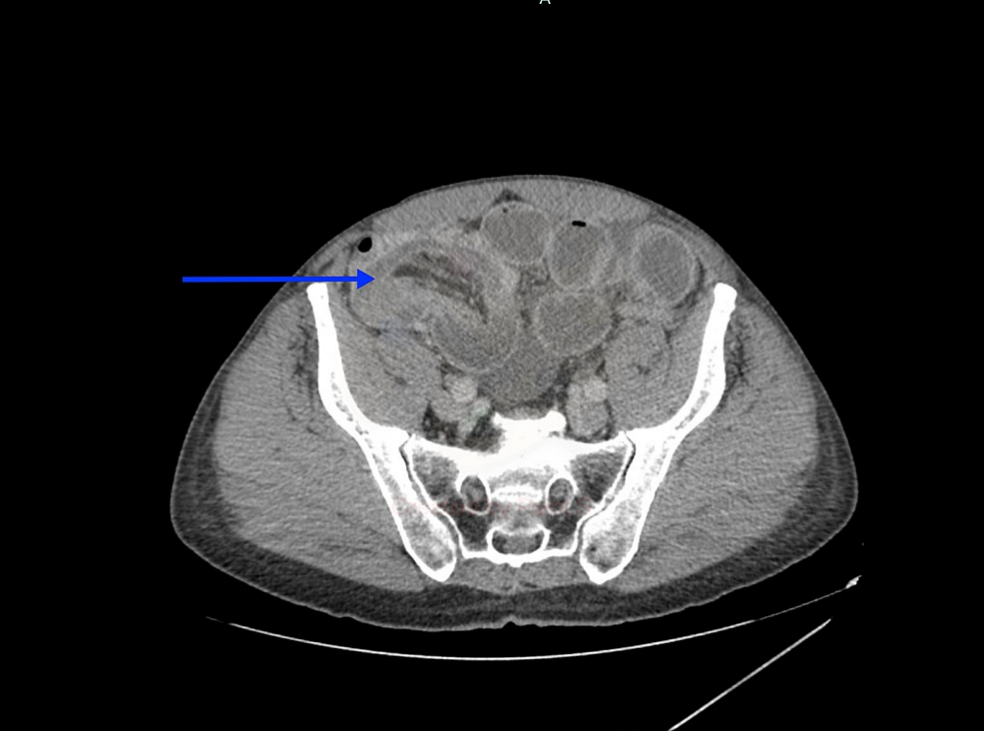
CT abdomen (axial view) showing intussusception of the distal ileum into cecum (blue arrow).

A 61-year-old male with no significant past medical or surgical history presented to our emergency department (ED) for evaluation of abdominal pain that had been going on for three days. On admission, he had 10/10 epigastric abdominal pain that radiated to his chest and to his legs, with hematochezia, nausea, and non-bloody vomiting. Physical exam of the patient revealed normal vital signs, a mildly distended and diffusely tender abdomen but no rebound tenderness, guarding or palpable masses. His initial labs showed normal electrolytes with a normal coagulation profile. His white cell count was 6.8 x 109/L, hemoglobin was 17.1 g/dL, and the platelets were 227 x 109/L. An emergent CT scan of the abdomen with contrast revealed a distended small bowel with an air-fluid level, mesenteric lymphadenopathy, and intussusception of the distal ileum into the cecum with obstruction (Figures 1-2)..

The patient had an emergent exploratory laparotomy, which showed a dilated segment of the small bowel along with ileocolic intussusception. The manual reduction was initially attempted which failed to resolve the intussusception, so a decision was made to undergo an ileocolic resection. Immunohistochemical stains performed on the terminal ileum and the regional lymph nodes showed the following results: CD20 positive, CD79A positive, CD10 positive, CD23 positive, BCL-2 positive, and BCL-6 positive. CD3 and CD5 showed scattered residual reactive T lymphocytes and CD138 showed scattered residual plasma cells, which were polytypic by Kappa and Lambda light chains.

**Figure 3:**
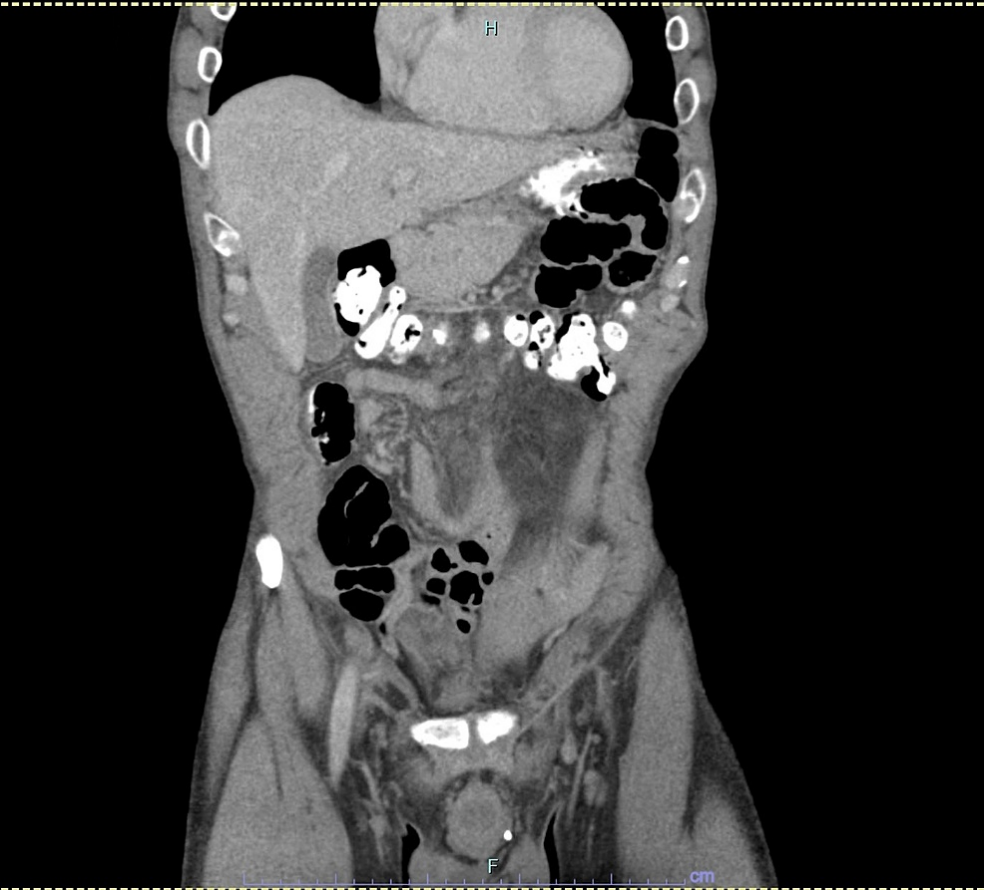
Abdominal CT scan after resection and chemotherapy showing resolution of small bowel obstruction and lymphadenopathy.

Bone marrow aspiration and biopsy were also done which showed no evidence of lymphoma. A subsequent positron emission tomography (PET)/CT scan showed intense uptake in the residual cecum and ascending colon (standardized uptake value, SUV 10.5); multiple mesenteric/peritoneal lymph nodes with the most prominent uptake adjacent to the cecum; and two left inguinal lymph nodes with intense uptake (SUV 6.7). A final diagnosis of follicular lymphoma, WHO grade 1-2, stage 2E was made, and the patient was started on the R-CVP regimen (cyclophosphamide, vincristine, and prednisone plus rituximab). He finished six cycles of immunochemotherapy. A subsequent CT scan of the abdomen and pelvis showed no lymphadenopathy (Figure 3). He is now being followed up in the Oncology clinic.

## Discussion

Adult intussusception can be broadly classified into four major categories based on their location: entero-enteric, ileo-cecal, ileo-colic, and colo-colic [3]. Pediatric cases of intussusception present with sudden onset of intermittent colicky pain, vomiting, bloody currant jelly stools, and the presence of a palpable mass. Contrary to that, adult cases can present with acute, subacute, or chronic non-specific symptoms [4]. The most common presenting symptom in adults is abdominal pain, found in 70%-100% of the cases. Other non-specific symptoms relate to obstruction and include nausea, vomiting, constipation, and weight loss [1-2].

One of the more uncommon causes of adult intussusception is GI lymphoma. Out of all the subtypes of lymphomas, it is the diffuse large B-cell variety that is implicated in most cases of intussusception [3]. Even though follicular lymphoma is the second most common subtype of lymphoma, it very rarely causes intussusception. One reason for this discrepancy is because follicular lymphomas only constitute 4% of all GI lymphomas [5-6]. To the best of our knowledge, there have only been a few such cases of follicular lymphomas with the initial presentation of intussusception.

Intestinal follicular lymphomas are rare and indolent. Most of the time, they are discovered incidentally during screening tests such as endoscopy, where they present as mucosal polyps [2, 5]. Unlike other forms of GI lymphomas, the indolent nature of these follicular lymphomas renders them hard to detect. This can result in some of these lymphomas presenting with intussusception as their initial finding. 

The treatment of adult intussusception is an area of controversy. Unlike children, intussusception in adults cannot be reduced by a non-surgical approach and a surgical intervention is always necessary [4, 7]. The debate lies in whether to perform an initial en-bloc resection versus surgical reduction and then resection [3-4]. Since the risk of malignancy associated with adult intussusception is so high, opting against a complete resection creates a potential for intraluminal seeding or venous tumor dissemination during manipulation [3-4, 8]. On the other hand, it is also true that initial reduction before resection can save considerable sections of the bowel and prevent the development of short bowel syndrome. Most authors agree that the involvement of the small bowel can be managed by initial reduction as the prevalence of a malignant cause is lower (30%), however, intussusception in the large bowel should be managed by en-bloc resection as the chance for malignancy is significantly higher (60%) [1, 3-4, 8].

 Chemoimmunotherapy, monotherapy with rituximab, radiotherapy, and surveillance are some of the established treatment modalities for follicular lymphomas. The choice to select between them is motivated by disease burden and the stage of the disease [9-11]. Since our patient had a high disease burden at presentation leading to intussusception and complete bowel obstruction, he was put on a chemoimmunotherapy regimen involving R-CVP as per the guidelines.

## Conclusions

Intussusception is an uncommon finding in adults and if present, should be carefully examined to rule out malignancy. Of all the malignant causes, follicular lymphoma is particularly rare, but as our report shows, it can still occur, and physicians should always be on the lookout for these rare and malignant causes. Due to the high likelihood of malignancy, all treatment options should be considered early in the disease process and the decision should be made after carefully weighing the pros and cons of each.
